# Polyunsaturated fatty acids alter the formation of lipid droplets and eicosanoid production in *Leishmania* promastigotes

**DOI:** 10.1590/0074-02760220160

**Published:** 2023-03-06

**Authors:** Yasmin Monara Ferreira de Sousa Andrade, Monara Viera de Castro, Victor de Souza Tavares, Rayane da Silva Oliveira Souza, Lúcia Helena Faccioli, Jonilson Berlink Lima, Carlos Arterio Sorgi, Valéria M Borges, Théo Araújo-Santos

**Affiliations:** 1 Fundação Oswaldo Cruz-Fiocruz, Instituto Gonçalo Moniz, Salvador, BA, Brasil; 2 Universidade Federal do Oeste da Bahia, Centro das Ciências Biológicas e da Saúde, Núcleo de Estudos de Agentes Infecciosos e Vetores, Barreiras, BA, Brasil; 3 Universidade Federal da Bahia, Faculdade de Medicina, Salvador, BA, Brasil; 4 Universidade de São Paulo, Faculdade de Ciências Farmacêuticas de Ribeirão Preto, Departamento de Análises Clínicas, Toxicológicas e Bromatológicas, Ribeirão Preto, SP, Brasil; 5 Universidade de São Paulo, Ribeirão Preto, SP, Brasil

**Keywords:** lipid droplet, eicosanoid, polyunsaturated fatty acids, GP63, PGF synthase, Leishmania

## Abstract

**BACKGROUND:**

The knowledge about eicosanoid metabolism and lipid droplet (LD) formation in the *Leishmania* is very limited and new approaches are needed to identify which bioactive molecules are produced of them.

**OBJECTIVES:**

Herein, we compared LDs and eicosanoids biogenesis in distinct *Leishmania* species which are etiologic agents of different clinical forms of leishmaniasis.

**METHODS:**

For this, promastigotes of *Leishmania amazonensis*, *L. braziliensis* and *L. infantum* were stimulated with polyunsaturated fatty acids (PUFA) and LD and eicosanoid production was evaluated. We also compared mutations in structural models of human-like cyclooxygenase-2 (GP63) and prostaglandin F synthase (PGFS) proteins, as well as the levels of these enzymes in parasite cell extracts.

**FINDINGS:**

PUFAs modulate the LD formation in *L. braziliensis* and *L. infantum*. *Leishmania* spp with equivalent tissue tropism had same protein mutations in GP63 and PGFS. No differences in GP63 production were observed among *Leishmania* spp, however PGFS production increased during the parasite differentiation. Stimulation with arachidonic acid resulted in elevated production of hydroxyeicosatetraenoic acids compared to prostaglandins.

**MAIN CONCLUSIONS:**

Our data suggest LD formation and eicosanoid production are distinctly modulated by PUFAS dependent of *Leishmania* species. In addition, eicosanoid-enzyme mutations are more similar between *Leishmania* species with same host tropism.

Lipid mediators are bioactive molecules derived from the metabolism of polyunsaturated fatty acids (PUFA).^1^ The most common lipid mediator precursors are derived from arachidonic (AA), eicosapentanoic (EPA), and docosahexaenoic (DHA) acids.^1^ Trypanosomatids, including *Leishmania*, can metabolise AA to eicosanoids by way of specific enzymes, such as cyclooxygenase (COX) and prostaglandin synthases (PG synthases).^2^ ^,^ ^3^ ^,^ ^4^ In addition, specialised lipid mediators are also identified in *Trypanosoma cruzi*.^4^ ^,^ ^5^ ^,^ ^6^

Lipid mediators are produced in the cytosol and in organelles termed lipid droplets (LD, lipid bodies),^7^ ^,^ ^8^ ^,^ ^9^ ^,^ ^10^ which are present in almost all organisms, including in trypanosomatid protozoa.^4^ ^,^ ^7^ ^,^ ^10^ ^,^ ^11^ ^,^ ^12^ LDs are active sites for eicosanoid metabolism.^7^ ^,^ ^8^ Although protozoan parasites are known to produce a variety of specialised lipid mediators, studies investigating the role of these mediators in parasite biology and host-parasite interaction remain scarce.

Parasites possess the necessary machinery to synthesise eicosanoids.^3^ *Leishmania* can metabolise AA to prostaglandins using PG synthases present in LDs.^7^ Recently, the glycoprotein of 63 kDa (GP63) was described as a Cox-like enzyme responsible to convert AA to prostaglandin in *L. mexicana.* ^2^ In addition to COX, trypanosomatids contain enzymes capable of synthesising other eicosanoids, such as PGE_2_ and PGF_2α_. Both *T. cruzi* trypomastigotes and *L. infantum* respond to exogenous AA stimulation by producing prostaglandins.^7^ ^,^ ^10^ However, the presence of AA was not shown to alter PGF synthase (PGFS) production in *L. infantum.* ^7^ *L. infantum* LDs are capable of synthesising PGF_2α_, a mediator responsible for increasing parasite viability in the initial moments of infection via an as yet unknown mechanism.^7^ *L. braziliensis* promastigotes and amastigotes also express PGFS, which may improve parasites fitness.^13^ Another pathway of lipid mediator production was recently described through the identification of proteins (CYP1, CYP2 and CYP3) similar to cytochrome P450 (CYP450) in the genome of *L. infantum*, which appear to be responsible for specialised lipid precursors in this parasite species.^14^

Advances have been made in our understanding of the metabolism of bioactive lipids found in parasites, as well as mechanisms involving LDs. Leishmaniasis presents a diversity of clinical forms and symptoms related to specific *Leishmania* species*.* Herein we compared the formation of LDs and the production of eicosanoids in different New World *Leishmania* species using PUFA precursors as stimulant. Differences were identified in lipid metabolism among the *Leishmania* species investigated, and the enzymes related to eicosanoid production were described.

## MATERIALS AND METHODS

*Parasites* - *L. infantum* (MCAN/BR/89/BA262) promastigotes were maintained for seven-nine days in hemoflagellate culture medium (HO-MEM) supplemented with 10% foetal bovine serum (FBS) until reaching stationary phase.^7^ For the cultivation of *L. amazonensis* (MHOM/BR/1987/BA125) and *L. braziliensis* (MHOM/BR/01/BA788), parasites were maintained in Schneider’s insect medium supplemented with 20% FBS, L-glutamine, 20 mM penicillin (100 U/mL) and streptomycin (0.1 mg/mL) at 26ºC for six days until reaching stationary phase.

*Stimulation of Leishmania* - *Leishmania* spp in the third day of axenic cultures (logarithmic-phase promastigote) were used to perform the experiments. In 96 wells plates, 1x10^6^ parasites/well were either treated with progressively higher doses of EPA, DHA (3.75, 7.5, 15, 30 µM) or AA (15 µM), or with ethanol 0,3% v/v (vehicle), or medium alone (control), for 1 h. The 15 uM dose for AA was used, as we previously verified that higher doses could interfere with the viability of the parasite.^7^ Next, parasites were fixed in formaldehyde 3.7% v/v and analysed by light microscopy as described below.

*Lipid droplet staining* - Fixed parasites were centrifuged on glass slides at 30g for 5 min. Slides were then washed with distilled water and subsequently kept in a 60% isopropanol solution for 5 min. Next, the slides were immersed in Oil Red O solution for 5 min. The slides were washed with distilled water and subsequently were mounted in aqueous medium, and the LDs marked by Oil Red O were quantified in 50 cells per slide using optical microscopy.^7^

**Fig. 1:**
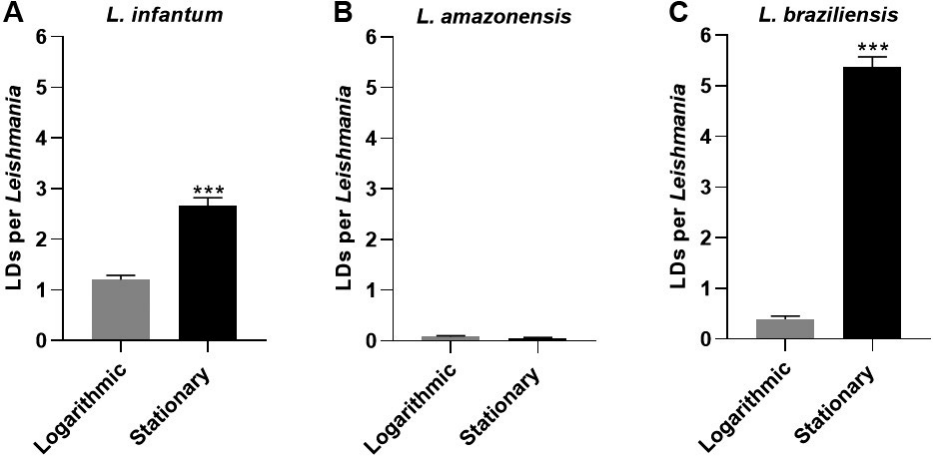
lipid droplets in procyclic forms of *Leishmania* spp. Parasites in logarithmic and stationary growth phases were labeled with Oil Red O to count lipid droplet (LD). Data shown represent the mean ± standard error of LDs in (A) *L. infantum*, (B) *L. amazonensis*, and (C) *L. braziliensis*.***, p < 0.0001 using Mann-Whitney test for multiple comparison by pairs.

*Parasitic viability* - Following treatment with PUFAs, *Leishmania* promastigotes were placed on 96-well flat-bottom plates in the presence of tetrazole salt (XTT) (ROCHE Applied Science) and incubated at 26ºC for 4 h in the dark. Next, XTT reduction by mitochondrial metabolism was evaluated by quantifying optical density on a plate reader (spectrophotometer) (Varioskan, ThermoScientific) [Supplementary data (Fig. 1)].

*Lipid extraction to identify PUFAs and eicosanoids in parasite cell extract* - After stimulation with AA, EPA or DHA, parasites isolated by centrifugation were subjected to hypotonic lysis in a 1:1 solution of deionised water and methanol at 4ºC. Culture supernatants were diluted at the same volume ratio in methanol at 4ºC. Samples were then stored at -80ºC until lipid extraction using liquid chromatography/mass spectrometry (LC/MS). Next, oxylipid extraction was performed using the solid phase extraction (SPE) method according to a previously described protocol.^15^ After lipid extraction, specimens were transferred to autosampler vials, and 10 μL of each sample were injected into the Nexera-TripleTOF^®^ 5600+ target liquid chromatography tandem mass spectrometry (LC-MS/MS) system (SCIEX, Foster City, California) as previously described by Sorgi et al.^15^ Final concentrations of oxylipids in culture extract and supernatants were quantified in accordance with standardised parameters.^15^

*In silico identification of enzymes involved in Leishmania eicosanoid metabolism* - At least two enzymes related to the production of lipid mediators have been identified in *Leishmania*: GP63 (human-like COX)^2^ and prostaglandin F synthase (PGFS).^4^ ^,^ ^7^ ^,^ ^16^ Initially, we performed a search for annotated nucleotide sequences characteristic of GP63 and PGFS using *L. major* as a reference species in GenBank. Using the BLASTn tool, complete genome sequences were identified and selected in the following species: *L. donovani*, *L. infantum*, *L. amazonensis*, *L. braziliensis*, *L. panamensis*, *L. mexicana*, *L. major* and *T. cruzi*. Only coding sequences (CDS) were considered for analysis [Supplementary data (Tables I-II)]. Multiple alignment of the obtained protein sequences was performed using the Clustal W (Codons) method. The molecular evolutionary genetics analysis (MEGA X) program was employed to construct a phylogenetic tree via the unweighted pair-group method with arithmetic mean (UPGMA) using 1000 bootstraps.^17^

*3D modeling of GP63 and PGFS proteins in Old and New World Leishmania species* - The protein data bank (PDB) was used to search for *L. major* crystallographic structures in order to identify the structures of the GP63 and PGFS proteins;^18^ PDB IDs: 1LML for GP63^19^ e PDB IDs: 4F40 for PFGS.^20^ The prediction of protein structures was then performed in other *Leishmania* spp using the Interactive Threading assembly refinement (I-TASSER) bioinformatics method.^21^ Using these structures, the PyMOL program was employed to create overlapping models and analyse the active site residues of these enzymes.^22^

*Western blot* - To perform protein extraction, parasite cell cultures in either stationary or logarithmic growth stages were centrifuged at 1620 *g* for 10 min, 4ºC. Next, the pellet at a concentration of 2x10^8^/mL was lysed using in 100 µL of radio immunoprecipitation buffer (RIPA). Next, the total amount of protein was quantified using the Pierce BCA protein assay (Thermo Scientific). Total proteins (30 µg) were separated by electrophoresis on a 10% polyacrylamide sodium dodecyl sulphate-polyacrylamide gel electrophoresis (SDS-PAGE) and then transferred to nitrocellulose membranes. Membranes were blocked in Tris saline buffer (TBS) containing 0.1% Tween 20 (TT) plus 5% milk for 1 h, followed by incubation with *L. infantum* anti-PGFS (1:1000) overnight.^7^ The primary antibody was then removed, and the membranes were washed five times in TT followed by incubation with the secondary antibody (goat anti-mouse) (SeraCare’s KPL Catalog 074-1806) conjugated to peroxidase (1:5000) for 1h. Finally, the membranes were washed 5x again and then incubated with Western Blotting substrate (Thermo Scientific Pierce ECL, Amersham, UK).

*GP63 immunoassays* - Protein extracts of *Leishmania* in logarithmic and stationary phase were submitted to enzyme-linked immunosorbent assay (ELISA) to measure GP63 production. Briefly, 96-well immunoassay plates were sensitised with 30µg of *Leishmania* protein overnight at 4ºC. Then, nonspecific binding was blocked with 0.1% PBS Tween 20 (PBS-T) plus 5% milk for 2 h. After blocking, the plates were incubated with anti-GP63 (1:50) (Catalog #MA1-81830 Thermofisher) and incubated overnight at 4ºC. Next, the plates were washed with PBS-T and incubated with the secondary antibody (SeraCare’s KPL Catalog 074-1806) conjugated with peroxidase (1:2000) for 1 h at room temperature. Finally, the plates were incubated with 3,3’5,5’-Tetramethylbenzidine (TMB) for 30 min, after which the reaction was stopped using 3M HCl. Plates were read on a microplate reader (Molecular Devices Spectra Max 340PC) a wavelength of 450 nm.

*Statistical analysis* - Statistical analyses were performed using GraphPad-Prism v8.0 software (GraphPad Software, San Diego, CA-USA). All obtained data are represented as means ± standard error of the mean. Statistical analysis was performed using One-way analysis of variance (ANOVA) with post-test of linear trend when comparing dose-response data, while the Mann-Whitney test was employed to multiple comparison by pairs of groups, at a significance level of p < 0.05. All experiments were performed in triplicate.

## RESULTS

**Fig. 2:**
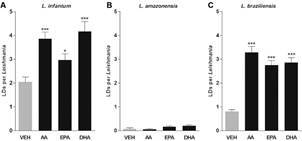
polyunsaturated fatty acids increase the formation of lipid droplets in procyclic forms of *Leishmania*. Logarithmic growth phase promastigotes of (A) *L. infantum* (B) *L. amazonensis* and (C) *L. braziliensis* were stimulated with ethanol (vehicle) or AA (15 µM), EPA (30 µM) or DHA (30 µM) for 1 h, and then stained with Oil Red O to quantify LDs. Bars represent means ± SEM of lipid droplets (LDs) per parasite. *** and * represent p < 0.0001 and p < 0.05, respectively, for multiple pairwise comparisons between stimuli and the vehicle using the Mann-Whitney test. AA: arachidonic acid; EPA: eicosapentaenoic acid; DHA: docosahexaenoic acid.

*Lipid droplets differ in quantity among Leishmania species* - Our previous work demonstrated increasing numbers of LDs as *L. infantum* parasites differentiated into metacyclic forms in vitro.^7^ Here, we also found higher numbers of LDs during the differentiation process in axenic cultures of *L. braziliensis* and *L. infantum*, but not in *L. amazonensis* (Fig. 1). In addition, LD formation was affected by stimulation with PUFAs; AA induced the formation of LDs in *L. braziliensis* and *L. infantum*, but not in *L. amazonensis* (Fig. 2). LD formation per parasite was found to be dose-dependent with regard to stimulation with EPA and DHA, as a statistically significant linear trend was observed for both *L. braziliensis* and *L. infantum*; however, no effect on LD formation was observed in *L. amazonensis* [Supplementary data (Fig. 2)]. Furthermore, the doses of PUFAs used in the tests did not affect the viability of the parasites [Supplementary data (Fig. 1)].

**Fig. 3:**
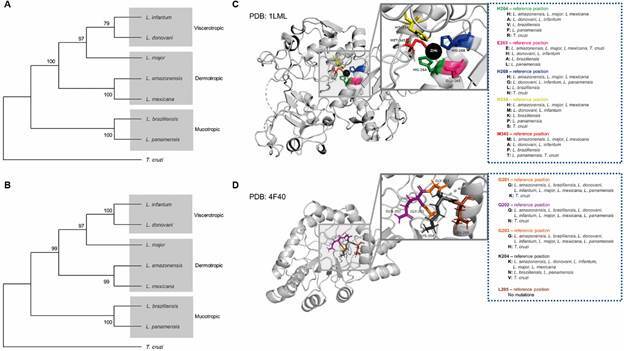
homology of GP63 and prostaglandin F synthase (PGFS) proteins across different *Leishmania* spp. *GP63* and *PGFS* genes were identified in the reference genomes of etiologic pathogens related to human leishmaniasis. Phylogenetic trees of (A) GP63 and (B) PGFS were constructed using the UPGMA method (MEGA X software), considering 1000 bootstraps. Residues at the active sites of (C) GP63 and (D) PGFS proteins are highlighted by colours depicted in three-dimensional structures. Non-synonymous mutations among *Leishmania* spp. are listed.

**Fig. 4:**
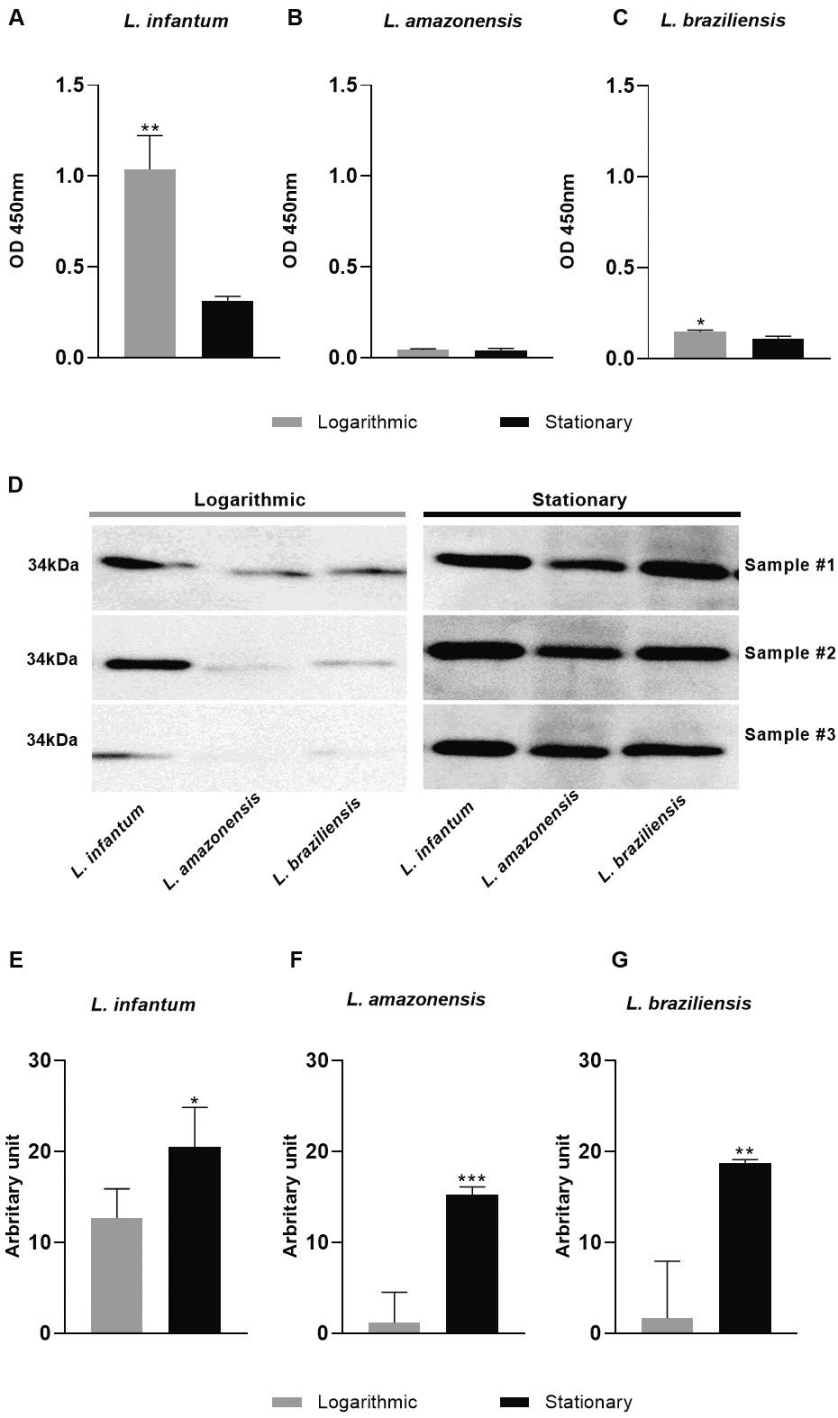
GP63 and prostaglandin F synthase (PGFS) protein production in logarithmic and stationary axenic stages of *Leishmania* spp. Parasites in logarithmic and stationary growth phases were lysed to measure GP63 protein production in (A) *L. infantum*, (B) *L. amazonensis*, (C) *L. braziliensis* by immunoassay. Total protein (30 µg) was incubated with anti-PGFS (1:500) for Western blot analysis. (D) Immunoblot comparing the abundance of PGFS in logarithmic and stationary stages incubated with anti-PGFS (1:1000) in (E) *L. infantum*, (F) *L. amazonensis*, (G) *L. braziliensis*. * p < 0.05 for multiple pairwise comparisons between stimuli and the vehicle the Mann-Whitney test.

**Fig. 5:**
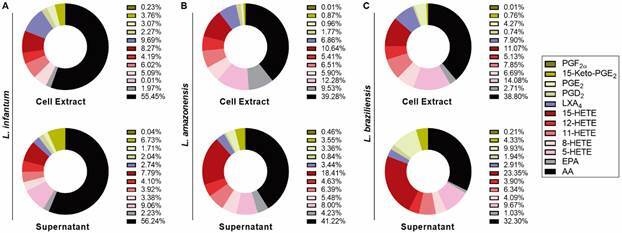
identification of lipid mediators in different *Leishmania* spp. Average distribution of the 10 abundant eicosanoids in the cell extract and supernatant of procyclic promastigotes of (A) *L. infantum*, (B) *L. amazonensis* and (C) *L. braziliensis* in logarithmic growth phase (with cell types labeled above each pie chart) stimulated with AA for 1 h, displayed as parts of the whole. Data are in percentage of ηg/mL of total lipid detected by liquid chromatography/mass spectrometry (LC/MS). EPA: eicosapentanoic acid; 15-HETE: 15-hydroxyeicosatetraenoic acid; 8-HETE: 8-hydroxyeicosatetraenoic acid; 11-HETE: 11-Hydroxyeicosatetraenoic acid; 12-HETE: 12-hydroxyeicosatetraenoic acid; AA: arachidonic acid; PGF_2α_: prostaglandin F_2α_; 15-keto-PGE_2_: 15-keto-prostaglandin E_2_; LXA_4_: Lipoxin A_4_; PGD_2_: prostaglandin D_2_; 5-HETE: 5-hydroxyeicosatetraenoic acid.

*Comparison of eicosanoid metabolism enzymes in Old and New World Leishmania spp* - Proteins associated with eicosanoid metabolism are present in *Leishmania* spp., such as a COX-like enzyme, previously known as GP63, and PGFS.^4^ We performed a comparison of the primary protein structure sequences in silico [Supplementary data (Fig. 3)]. In addition, the tertiary structures of these reference proteins exhibited high structural similarities across *Leishmania* spp, as well as *T. cruzi* [Supplementary data (Figs 4-5)]. Alignment and phylogenetic analysis of GP63 and PGFS indicated notable similarity and homology between the Old and New World *Leishmania* spp analysed with respect to clinical manifestations of disease [viscerotropic, dermotropic or mucotropic; Supplementary data (Fig. 3), Figs 3A-B]. In addition, nonsynonymous mutations were identified in the amino acid residues at the active sites of GP63 (PDB ID: 1LML) and PGFS (PDB ID: 4F40). *L. infantum* and *L. donovani* shared mutations in GP63 protein residues. The dermotropic species presented same residues in the active site of the GP63 enzyme, while the species with mucosal tropism presented exclusive mutations in the active site of GP63 (Fig. 3C). Furthermore, most of the PGFS residues among viscerotropic, dermotropic and mucotropic species were conserved, except for the K204 residue that was altered in *L. braziliensis* and *L. panamensis*, both mucotropic species (Fig. 3D).

We then compared the gene expression of these two enzymes involved in the production of eicosanoids in different *Leishmania* species. GP63 was more produced in log-stage *L. infantum* and *L. braziliensis* parasites, but not in *L. amazonensis* (Fig. 4A-C). In addition, PGFS was more produced in stationary parasites compared to log-stage procyclic forms in all three species of *Leishmania* (Fig. 4D-G).

*Qualitative effects of PUFAs on eicosanoid production in Leishmania spp* - Trypanosomatids possess enzymatic machinery for the metabolisation of PUFAs to specialised and conventional lipid mediators.^4^ ^,^ ^5^ ^,^ ^14^ Herein we used LC/MS to evaluate the presence of lipid mediator precursors, as well as eicosanoids, in *L. infantum*, *L. amazonensis* and *L. braziliensis* treated or not with AA, EPA or DHA. In all, 41 lipid mediators were analysed in cell extracts and axenic culture supernatants. The presence of twelve bioactive lipids was identified: 15-keto-PGE_2_, LXA_4_, PGD_2_, PGE_2_, 5-HETE, AA, 12-HETE, 8-HETE, 11-HETE, EPA, 15-HETE, PGF_2α_ (Table and Fig. 5). However, mediators LTC_4_, PGB_2_, 6-keto-PGF1a, 17-RvD1, 12-oxo-LTB_4_, 20-OH-PGE_2_, TXB_2_, RvD1, RvD2, RvD3, LTB_4_, LTD_4_, LTE_4_, 6-trans-LTB_4_, 11-trans-LTD_4_, PDx, Maresin, PGJ_2_/PGA_2_, RvE1, 15-deoxy-PGJ_2_, 5-oxo-ETE, 20- HETE, 5,6-DiHETE, 12-oxo-ETE, 15-oxo-ETE, 11,12-DiHETrE, 14,15-DiHETrE, 5,6- DiHETrE, 20-OH-LTB4 were not identified. As expected, AA-derived eicosanoids were the most prevalent in *Leishmania* extracts (Fig. 5). In addition, eicosanoid production was poorly detected when parasites were stimulated with EPA and DHA (data not shown).

**TABLE:** Quantitation of eicosanoids and their precursors in cell extract and supernatant of *Leishmania* spp. by liquid chromatography/mass spectrometry (LC/MS)

Lipids	Standards^*^	Mass (m/z)		Lipid concentrations in cell extract and supernatant by *Leishmania* spp (ηg/mL)*					
		Precursor ion (m/z)	Fragment ion (m/z)	*L. infantum*		*L. amazonensis*		*L. braziliensis*	
				C	S	C	S	C	S
PGF_2α_	PGF_2α_-d4	353	309.2179	0.17	0.27	< 0.01	4.68	< 0.01	1.62
15-Keto-PGE_2_	PGE_2_-d4	349	287.2017	2.82	43.80	1.32	36.12	1.12	33.89
PGE_2_	PGE_2_-d4	351	189.1285	2.30	11.14	1.46	34.19	6.31	77.74
PGD_2_	PGD_2_-d4	351	189.1285	1.70	13.29	2.69	8.56	1.09	15.21
LXA_4_	LXA_4_-d5	351	217.1598	7.27	17.59	10.46	35.05	11.66	22.76
15-HETE	15-HETE-d8	319	175.1492	6.20	50.66	16.23	187.49	16.34	182.75
12-HETE	12-HETE-d8	319	179.1078	3.14	26.65	8.26	47.16	7.57	30.52
11-HETE	12-HETE-d8	319	167.1084	4.52	25.52	9.93	65.03	11.59	49.60
8-HETE	12-HETE-d8	319	155.0714	3.82	22.01	9.00	55.83	9.88	32.00
5-HETE	5-HETE-d8	319	115.0401	< 0.01	58.94	18.74	81.46	20.79	75.68
EPA	AA-d11	301	257.2275	1.48	14.54	14.54	43.07	3.99	8.10
AA	AA-d11	303	259.2447	41.59	365.81	59.94	419.78	57.27	252.77

AA: arachidonic Acid; EPA: eicosapentaenoic acid; LX: lipoxin; PG: prostaglandin; HETE: hydroxyeicosatetraenoate; C: cell extract; S: supernatant. *Standard molecules containing Deuterium (d) atoms was used as an internal standard for the quantification of lipids by LC-mass spectrometry (MS).

## DISCUSSION

*Leishmania* lipid droplets are sites of production of eicosanoids, which are important for modulating the response to the parasite in vitro. However, the literature contains scarce data on eicosanoid metabolism in these parasites.^4^ Here we compared eicosanoid metabolism and LD formation in response to PUFAs in different species of *Leishmania* associated with distinct clinical forms. Our data show that, in contrast to *L. amazonensis*, LD formation can be modulated by PUFA stimulation in *L. infantum* and *L. braziliensis.* However, with regard to eicosanoid production, the same eicosanoids were detected across all three New World species when stimulated with AA. The mechanisms of regulation of the production of LDs in *Leishmania* are still poorly understood. *Trypanosomatids* have only two proteins related to the LD formation, the lipid droplet kinase (LDK) and *Trypanosoma brucei* Lipin (TbLpn).^4^ ^,^ ^23^ ^,^ ^24^ More studies will be necessary to investigate the presence and function of both LDK and TbLpn in *Leishmania* species. We can hypothesise that decrease in the activity in *L. amanozensis* could explain the inability of this parasite species to respond to PUFAs stimuli with the formation of LDs.

LDs are organelles that synthesise lipid mediators in a variety of cell types.^8^ However, the role of LDs in eicosanoid production in parasites remains poorly understood.^4^ In *L. infantum*, LDs are sites of production for PGF_2α_,^7^ but the literature contains scare reports on the presence of enzymes related to the lipid metabolism of eicosanoids or bioactive lipids in other protozoa. Herein, we found that AA modulated the formation of LDs, which suggests the accumulation of AA in LDs that may serve as a platform for the synthesis of eicosanoids in some protozoa of the genus *Leishmania*.

Regarding the formation of lipid mediators, it is known that enzymatic machinery in protozoa is responsible for the synthesis of these bioactive compounds, such as GP63^2^ and PGFS.^4^ ^,^ ^7^ ^,^ ^10^ ^,^ ^13^ ^,^ ^16^ However, the enzymes that convert AA into lipid mediators have not been adequately studied. In *L. mexicana*, a GP63 protein was shown to be analogous to COX-2.^2^ These protein metalloproteases are described as the main surface antigen expressed in promastigotes of different *Leishmania* species.^25^ Although the genes encoding the GP63 metalloproteases are organised in tandem,^26^ ^,^ ^27^ it has not been demonstrated whether COX-2 activity would arise from all of these encoded proteins. The production of PGF2α, which enzyme PGFS is responsible for synthesising,^3^ activates the PGF2α receptor, triggering the COX pathway.^28^ While little is known about the role of parasite-derived PGFS, some studies suggest the potential role of this eicosanoid in host-parasite interaction.^7^ ^,^ ^13^ The analysis of the viscerotropic and dermotropic *Leishmania* species point out some genetic differences related to parasite tropism.^29^ However, a relationship between the genetics of the parasites and the pathophysiology is still necessary. In this sense, identifying the differences in the production of eicosanoids can help to explain the distinct clinical manifestations caused between different species of *Leishmania*, since the mechanisms of eicosanoid action during inflammation are well knowns.^8^ ^,^ ^30^ Our comparison of protein sequences and GP63 and PGFS active site residues in both Old and New World *Leishmania* species revealed surprising similarity between these enzymes in accordance with the clinical form of disease caused by the parasite. Nonetheless, additional studies are needed to verify if, in fact, this similarity could be related to GP63 and PGFS production, and to the development of a polarised response according to the clinical manifestation. Indeed, some studies in humans have shown altered production of lipid mediators depending on the cutaneous or visceral form of disease.^7^ ^,^ ^31^

An important aspect of our evaluation focused on the production of enzymes involved in the metabolism of eicosanoids during the metacyclogenesis of different New World *Leishmania* species. The COX-like enzyme GP63 production reduces during *L. braziliensis* and *L. infantum* promastigotes differentiation, but no differences are observed in *L. amazonensis*. In the next AA metabolism step, PGFS production increases during parasite differentiation in different *Leishmania* species. This finding may indicate that PGFS could influence the virulence of infective forms of *Leishmania* spp. Considering the differences in the structure of and family of genes encoding these enzymes, further studies are needed to correlate these differences with the enzymatic activity exhibited by PGFS in *Leishmania*.

While some studies have advanced the understanding of lipid metabolism, the enzymes involved in parasites remain poorly described; however, knowledge on which metabolites are produced by zoonotic parasites is expanding.^2^ ^,^ ^9^ ^,^ ^10^ ^,^ ^14^ Here, we investigated the metabolites produced by different *Leishmania* species, observing increased production of lipid mediators of the HETE class. Importantly, little is known about the role played by these metabolites during the host-parasite interaction process. Additional studies may shed light on whether these should be considered virulence factors and thus may serve as intervention targets, in addition to whether their currently unidentified receptors could elucidate mechanisms of pathogenicity. The present findings serve to open perspectives by providing evidence on how PUFAs lead to the modulation of LD formation in different Old and New World *Leishmania* species. We believe that our qualitative overview of lipid mediators potentially produced by these parasites contributes to the base of knowledge surrounding antiparasitic drug development.
