# Supplementary material for: Polyunsaturated fatty acids alter the formation of lipid droplets and eicosanoid production in Leishmania promastigotes
Source: Mem Inst Oswaldo Cruz. 2023 Mar 6;118:e220160. doi: 10.1590/0074-02760220160 (PMC9991015; doi:10.1590/0074-02760220160)
Supplement: Supplementary file 1 [file 1678-8060-mioc-118-e220160-s.pdf]

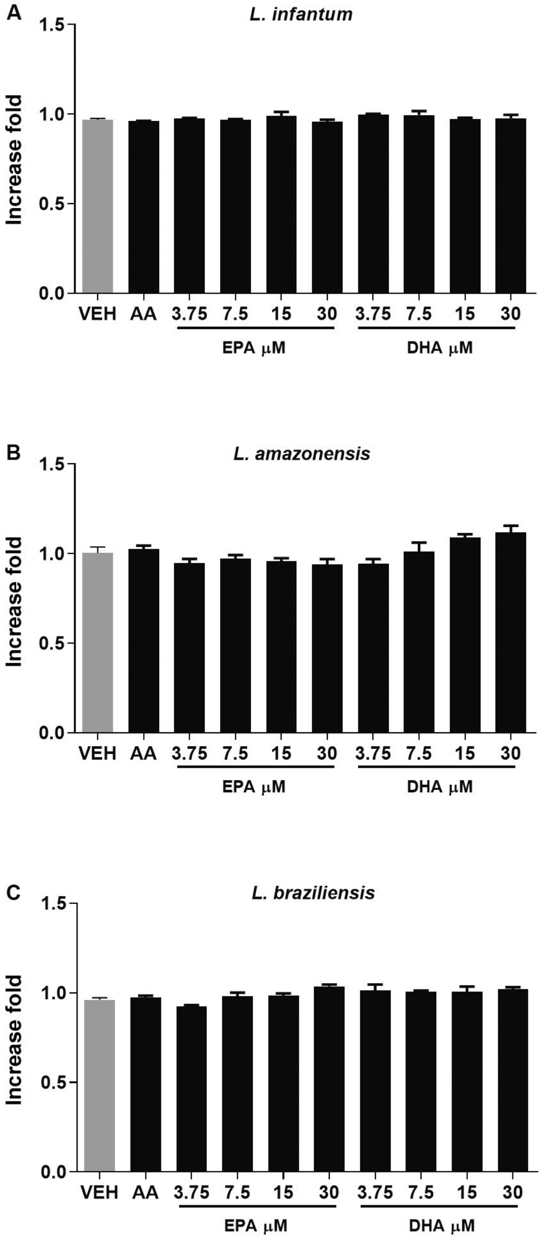

Fig. 1: polyunsaturated fatty acids stimulation does not affect *Leishmania* viability. Procyclic promastigotes of (A) *L. infantum*, (B) *L. amazonensis* and (C) *L. brasiliensis* in logarithmic growth phase were stimulated with AA, EPA or DHA for 1 h. Next, tetrazolium salt (XTT) reduction was measured by spectrophotometry. Data are represented as means ± standard error of optical density readings.

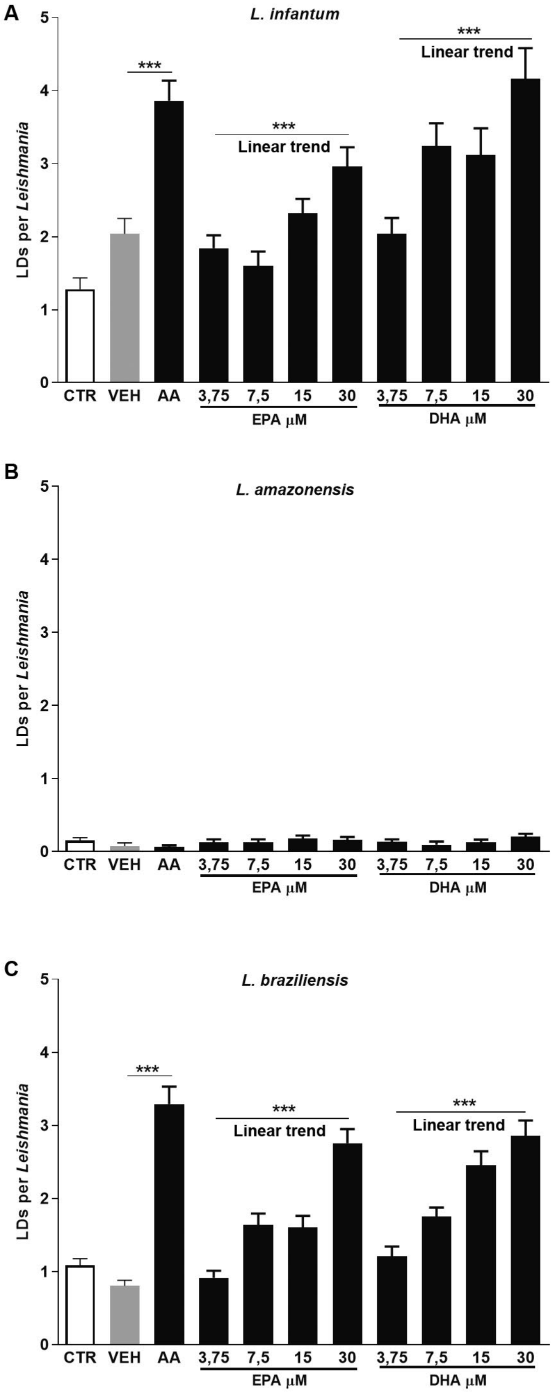

Fig. 2: polyunsaturated fatty acids increase the formation of lipid droplets in procylic forms of *Leishmania*. Logarithmic growth phase promastigotes of (A) *L. infantum* (B) *L. amazonensis* and (C) *L. braziliensis* were stimulated with ethanol (vehicle) or AA (15 µM), EPA (3.75, 7.5, 15 or 30 µM) or DHA (3.75, 7.5, 15 or 30 µM) for 1 h, and then stained with Oil Red O to quantify LDs. Bars represent means ± SEM of LDs per parasite. *** represent $p < 0.0001$, for pairwise comparison between AA and the vehicle using the Mann-Whitney test. The significance was tested by One-way ANOVA with post-test linear trend to dose response stimuli. AA: arachidonic acid; EPA: eicosapentaenoic acid; DHA: docosahexaenoic acid.

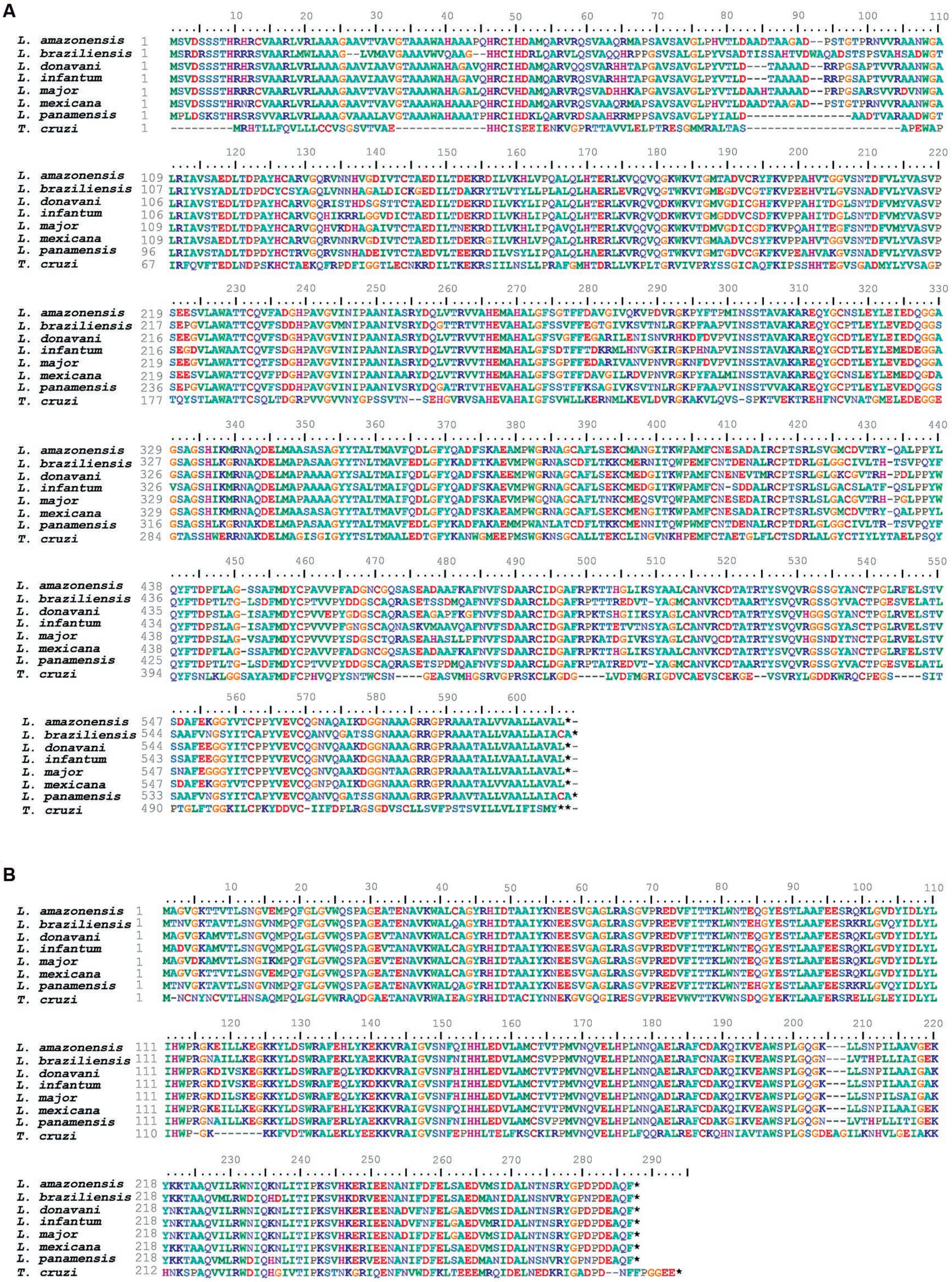

Fig. 3: comparative analysis of the primary structure of GP63 and PGFS proteins across New and Old World *Leishmania* spp. Protein sequences of (A) GP63 or (B) PGFS were aligned using the ClustalW algorithm.

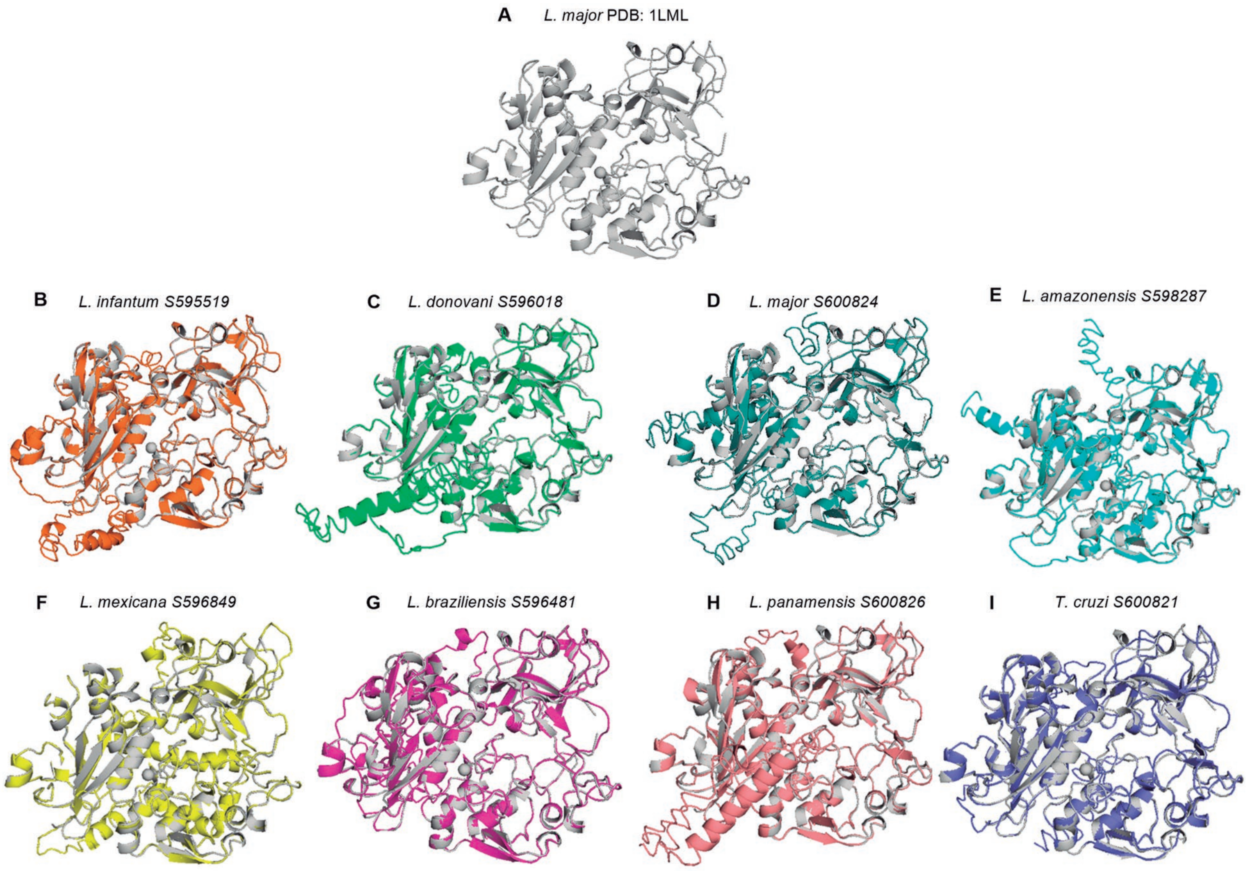

Fig. 4: comparative analysis of the tertiary structure of GP63 protein in *Leishmania* spp. and *Trypanosoma cruzi.* The GP63 protein was modeled using the I-TASSER algorithm and structures were aligned over the *L. major* protein (grey). GP63 tertiary structure overlap shows similarities between (A) *L. major* PDB: 1LML and (B) *L. infantum*, (C) *L. donovani*, (D) *L. major*, (E) *L. amazonensis*, (F) *L. mexicana*, (G) *L. braziliensis*, (H) *L. panamensis* and (I) *T. cruzi.*

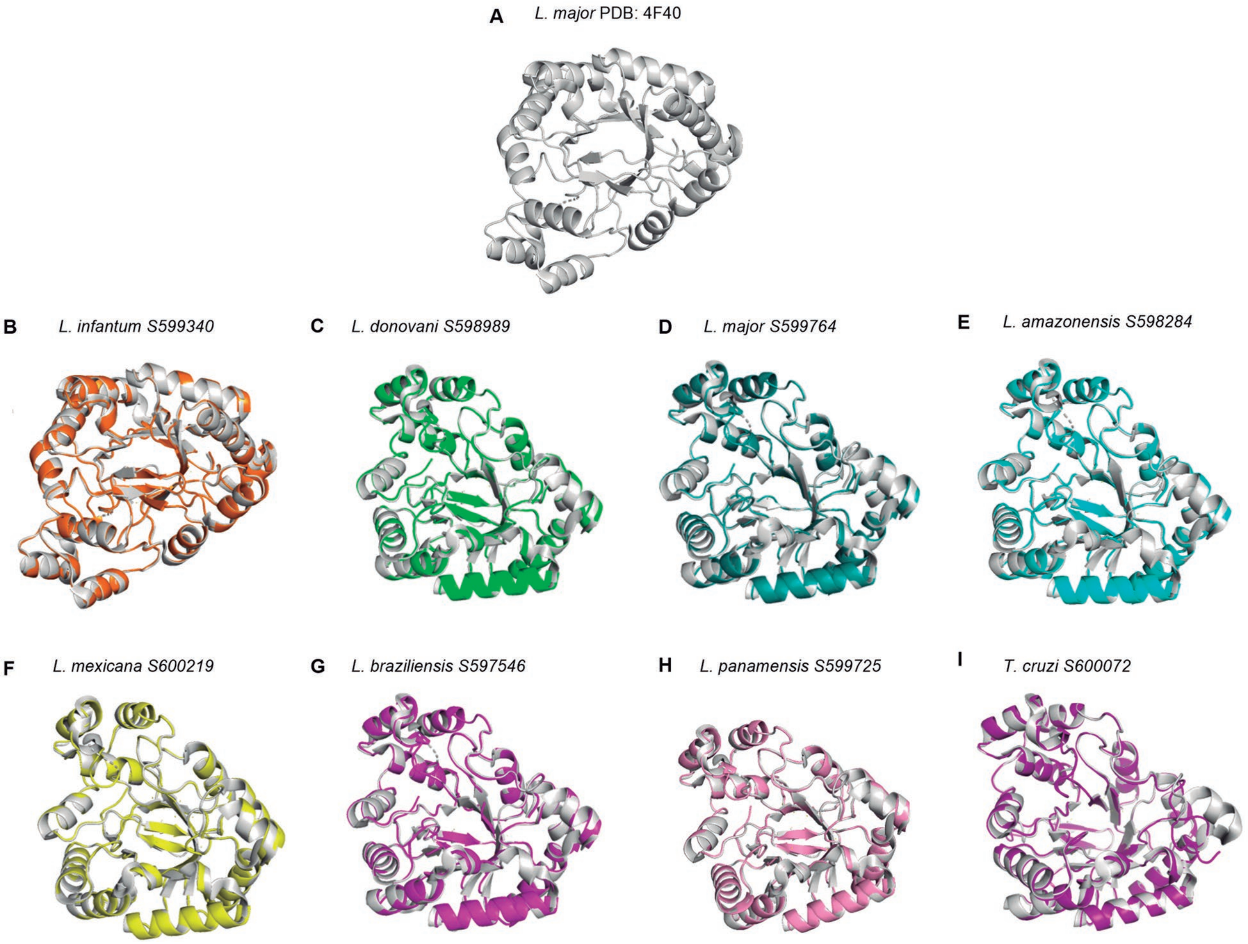

Fig. 5: comparative analysis of the tertiary structure of PGFS protein in *Leishmania* spp. and *Trypanosoma cruzi.* The PGFS protein was modeled using the I-TASSER algorism and structures were aligned over the *L. major* protein (grey). PGFS tertiary structure overlap shows similarities between (A) *L. major* PDB: 4F40 and (B) *L. infantum*, (C) *L. donovani*, (D) *L. major*, (E) *L. amazonensis*, (F) *L. mexicana*, (G) *L. braziliensis*, (H) *L. panamensis* and (I) *T. cruzi*.

TABLE I

GP63 nucleotide sequences used in the study

Species	GenBank code	region (begin - end)	Size (bp)	UniprotKB code
Leishmania infantum	FR796442.1	222401 - 224197	1800	Q6LA77
Leishmania donovani	CP029509.1	257944 - 259743	1800	A0A3S7WR60
Leishmania major	Y00647.1	199 - 2007	1809	P08148
Leishmania amazonensis	CP040138.1	179953 - 181761	1809	No annotation
Leishmania mexicana	NC_018314.1	180317 - 182125	1809	E9AN54
Leishmania braziliensis	LS997609.1	224752 - 226554	1803	A0A3P3YZR7
Leishmania panamensis	AF037166.1	1 - 1770	1770	O46312
Trypanosoma cruzi	MKQG01002498.1	73636 - 75255	1630	No annotation

TABLE II

PGFS nucleotide sequences used in the study

Species	GenBank code	region (begin - end)*	Size (bp)	UniProtKB code
Leishmania infantum	FR796463.1	1034722 - 1035576	855	A4I6Z4
Leishmania donovani	FR799618.2	1062787 - 1063641	855	E9BMZ2
Leishmania major	FR796427.1	1050960 - 1051814	855	P22045
Leishmania amazonensis	CP040158.1	1024015 -1023161	855	No annotation
Leishmania mexicana	FR799583.1	1026690 -1027544	855	E9B215
Leishmania braziliensis	FR799006.1	1114033 - 1114887	855	A4HJJ7
Leishmania panamensis	CP009400.1	936089 - 936943	855	A0A088RXB1
Trypanosoma cruzi	AAHK01000429.1	11770 - 12618	849	Q4DJ07

*gene identified in the reverse complementary sequence
